# Evolvement of Uniformity and Volatility in the Stressed Global Financial Village

**DOI:** 10.1371/journal.pone.0031144

**Published:** 2012-02-08

**Authors:** Dror Y. Kenett, Matthias Raddant, Thomas Lux, Eshel Ben-Jacob

**Affiliations:** 1 School of Physics and Astronomy, Tel-Aviv University, Tel-Aviv, Israel; 2 Kiel Institute for the World Economy, Kiel, Germany; 3 Department of Economics, University of Kiel, Kiel, Germany; 4 Bank of Spain Chair, University Jaume I, Castellón, Spain; Universidad Veracruzana, Mexico

## Abstract

**Background:**

In the current era of strong worldwide market couplings the global financial village became highly prone to systemic collapses, events that can rapidly sweep throughout the entire village.

**Methodology/Principal Findings:**

We present a new methodology to assess and quantify inter-market relations. The approach is based on the correlations between the market index, the index volatility, the market Index Cohesive Force and the meta-correlations (correlations between the intra-correlations.) We investigated the relations between six important world markets—U.S., U.K., Germany, Japan, China and India—from January 2000 until December 2010. We found that while the developed “western” markets (U.S., U.K., Germany) are highly correlated, the interdependencies between these markets and the developing “eastern” markets (India and China) are volatile and with noticeable maxima at times of global world events. The Japanese market switches “identity”—it switches between periods of high meta-correlations with the “western” markets and periods when it behaves more similarly to the “eastern” markets.

**Conclusions/Significance:**

The methodological framework presented here provides a way to quantify the evolvement of interdependencies in the global market, evaluate a world financial network and quantify changes in the world inter market relations. Such changes can be used as precursors to the agitation of the global financial village. Hence, the new approach can help to develop a sensitive “financial seismograph” to detect early signs of global financial crises so they can be treated before they develop into worldwide events.

## Introduction

Has the world become one small financial global village? Coupling between the world's different markets has become stronger and stronger over the past years, as is evidenced by the financial difficulties, which are affecting many markets around the globe, especially since late 2008. The growing financial integration allows capital to flow rather freely between countries and markets. Investments in stocks can be diversified into global portfolios, consisting of multiple assets from a large number of markets. As a result, stock markets have turned into an extended and strongly coupled complex system, in which large movements in price and volatility are likely to be transferred from one market to the other due to portfolio readjustments. Engle et al. [1] have shown that volatility clusters are likely to occur jointly in different markets. This fact and other evidence of the interdependencies between the world's economies emphasize the need to understand the coupling and integration of stock markets around the world. As the financial crisis of 2008 was not even considered a possibility by the leading economic theories [2], it is necessary to rethink and reformulate the understanding and quantification of the coupling between different markets.

When it comes to the analysis of individual markets, a wealth of different measures have been devised and used to analyze similarity between financial time series. These include Pearson's correlations [3]–[6], co-movement measures [7], recurrence patterns [8], and regime switching approaches [9], [10]. There are also studies of the co-movement of different stock markets. Forbes and Rigobon [11] have shown that a high level of dependence is visible between most markets and that changes in correlation are coupled to volatility changes. However, there are mixed results about the driving forces of the amount of co-movement and of financial integration. While King et al. [12] did not clearly identify the reasons for changes in the correlation, Beile and Candelon [13] found evidence that increased trade and financial liberalization go hand in hand with a synchronization of stock markets. Furthermore, Ahlgren and Antell [14] found that markets are linked closer in times of crisis which significantly hampers the possibility to diversify investments and thus risks. Additional studies looked at correlation structures in particular markets, like Tumminello et al. [15], or at the correlation between the indices of different markets, see e.g. Song et al. [16].

Recently, Kenett et al. investigated the dynamics of correlations between stocks belonging to the S&P 500 index, and the residual (partial) correlations after removing the influence of the index [3]. To this end, the Index Cohesive Force (ICF), which is the ratio between the average stock correlation, and average stock partial correlation, was introduced. Studying the dynamics of these quantitative measures, a transition in the dynamics of the U.S. market at the end of 2001 was observed. Here we expand these previous analyses to the investigation of other markets. We further extend the scope of the analysis by studying the markets intra and inter correlations. First, we study correlation structures on the level of single markets, the market intra-correlation. Next, we study the correlation between different market pairs, according to three measures - the market index correlation, market meta-correlation, and market ICF correlation.

## Materials and Methods

### Methods

The similarity between stock price changes is commonly calculated via the Pearson's correlation coefficient. The raw stock correlations [5] are calculated for time series of the log of the daily return, given by:
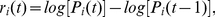
(1)where 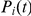 is the daily adjusted closing price of stock 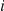 at day 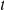. The raw stock correlations are calculated using Pearson's correlation coefficient between every pair of stocks 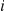 and 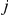, where
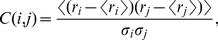
(2)

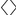
 denotes average, and 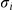 are the standard deviations (STD).

Partial correlation is a powerful tool to investigate how the correlation between two stocks depends on the correlation of each of the stocks with a third mediating stock or with the index as is considered here. The residual, or partial, correlation between stocks 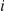 and 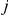, using the index 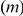 as the mediating variable is defined by [6], [17], [18].
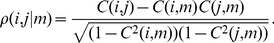
(3)


Note that according to this definition, 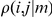 can be viewed as the residual correlation between stocks 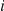 and 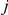, after subtraction of the contribution of the correlation between each of the stocks with the index.

To investigate the dynamics of correlations in capital markets, we make use of a running window analysis. We use a short time window, of 22-trading days, which is equivalent to one work month, with a full overlap. Thus, for example the first window will be days 1–22, the second window day 2–23, etc. At each window we calculate stock correlation and partial correlation matrices, and average them. This results in a value of correlation (partial correlation) for each stock, representing its average correlation (partial correlation) to all other stocks. This is defined as
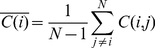
(4)

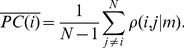
(5)


Finally, we calculate the average of average correlations (partial correlations), which represents the total average correlation (partial correlation) in the market,
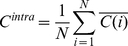
(6)

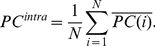
(7)


We denote this variable as the intra-correlation (intra partial correlation), as it represents the average correlation of stocks belonging to one given market.

Next, we investigate the synchronization of two given markets. To this end, we calculate correlation and lagged cross correlation between the intra correlations of each market. The correlation of market correlations is denoted as market meta-correlation (MC), given by
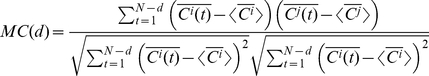
(8)

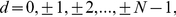
(9)where 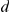 is the lag.

Recently, it was shown that the market index has a cohesive effect on the dynamics of the stock correlations [3], [6]. This refers to the observed effect the index has on stock correlations, where we have found that larger changes of the index result in higher stock correlations, and as such more cohesive force. The Index Cohesive Force is defined as 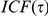 – calculated over a time window 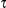, as a measure of the balance between the raw and residual correlations given by,
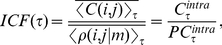
(10)where 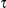 is the time window, during which the average correlation and average residual correlation are calculated. 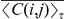 and 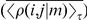 are the mean of average correlation and average partial correlation.

### Data

For the analysis reported in this paper we use data of the daily adjusted closing price from stocks in six different markets, all downloaded from Thomson Reuters Datastream. The markets investigated are the U.S., U.K., Germany, Japan, India and China. These include the four main stock markets as well as two less developed markets for comparison of the results. For each market we aimed for a sample as broad as possible, without any ex ante selection of branches. See Table 1 for details on the used stocks. The number of stocks finally used in our analysis shrinks down significantly, because we only consider stocks that are active from January 2000 until December 2010. Volume data was used to identify and eliminate illiquid stocks from the sample. Here, this corresponded to filtering for stocks which had no movement in the price for more than 6 percent of the 2700 trading days.

**Table 1:** Summary of data used.

Market	Stocks used	Index used	# before	# filtered
U.S.	S&P 500	S&P 500	500	403
U.K.	FTSE 350	FTSE 350	356	116
Germany	DAX Composite	DAX 30 Performance	605	89
Japan	Nikkei 500	Nikkei 500	500	315
India	BSE 200	BSE 100	193	126
China	SSE Composite	SSE Composite	1204	69

It should be noted that the correlations measured have some explanatory limitations, which are mainly due to structural differences of the markets and to selection issues of the stocks. First of all, the dataset is by construction biased towards long-lived stocks. Secondly, the intra-market correlations have been calculated on the basis of a market index, which composition is undergoing changes over time. However, we are pretty certain that the exact composition of the index used for the normalization does not have significant influence on the results.

When we compare time series from different markets, some adjustments must be made, mostly due to differences in trading days. To this end, we either only used data from days in which trading was done in both markets, or we replaced missing data with that of the observation of the last trading day. These two methods yield very similar results for the correlation analysis. When comparing all markets together, we used the joint trading period of the London (U.K.) and Frankfurt (Germany) stock exchange (the bilateral pair which has the most overlap with all other markets) and again replaced missing observations for all other markets with last day observation. For comparisons of the U.S. and Japan one should be aware that it makes sense to consider observations of day 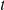 for the U.S. and 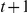 for Japan (the date barrier is in the Pacific), since these observations are closer to each other in terms of trading hours. Similar considerations can be taken for China and India, although the effect here is much weaker. In general the calculated correlations might also be influenced by the amount of overlap in daily trading hours, the amount of overlap in trading days, and general economic differences (as mentioned in the discussion). Also, the results depend on the time scale. Here we are interested in the medium run (a few weeks). A different kind of analysis of short-run effects, including a more detailed look on volatility, could be done with high-frequency (tick) data.

## Results

### Dynamics of the individual markets

**Figure 1:**
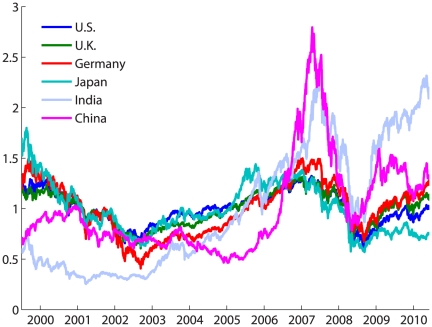
Normalized stock price indices; S&P 500 (U.S.), FTSE 350 (U.K.), DAX 30 Performance (Germany), NIKKEI 500 (Japan), BSE 100 (India), and SSE Composite (China). All indices have been normalized by their mean. The indices of the U.S., U.K. and Germany (blue, green and red line) appear almost as if they are shifted parallel, which is a sign of their high correlation. Note that all price indices are based on stock prices in local currency.

A first proxy to the dynamics of the different world's economies is the dynamics of their leading market indices. Here we focus on six of the world's largest economies, representing western markets – U.S., U.K., and Germany – and eastern markets - Japan, India and China. The stock price indices of these countries are presented in Figure 1, showing mostly very similar dynamics.

**Figure 2:**
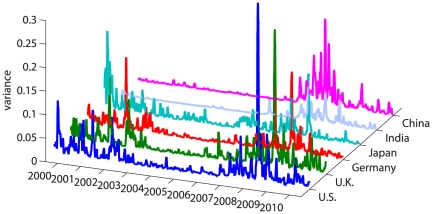
Relative volatility in the markets within a 22-day window. The price indices data was standardized for the 10-year interval (the mean is zero and the variance is 1 for each complete time series). The volatility peaks for the U.S, U.K. and Germany mostly coincide while there is less similarity with Japan. India and China show a very different behavior of volatility, especially until 2007.

Investigating the index volatility, rather than the index price reveals meaningful hidden information. Studying Figure 2, a similarity is observed between the three “western” markets, while the volatility peaks of the “eastern” markets only coincide for some time periods. Thus, it is reasonable to ask whether such uniformity between some markets, and multiformity between others, can be quantified.

### Dynamics of market intra-correlations

**Figure 3:**
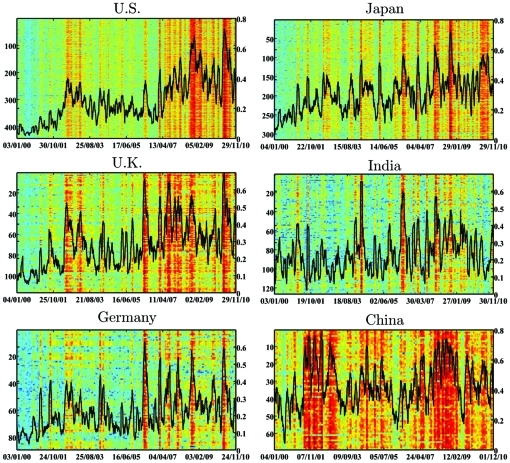
Dynamics of the intra-correlation. For each market, we use a 22-day window, and in each window calculate the intra-correlation. This results in the dynamics of the intra-correlation for the period of 2000–2010, for each market separately. Each horizontal line represents the average correlation of one stock (the left y-axis displays the number of the stock). The western markets and Japan show a similar behavior, visualized through vertical stripes at the same time, showing synchronized waves of strong correlations. The black line shows the average of all correlations at a given 22-day window (corresponding to the right y-axis). The trend is increasing for all countries except for China.

To understand the dynamics of capital markets, much research has focused on the analysis of correlations [5], [19]–[21]. It is standard practice to calculate the correlations between stocks in a given market, and we correspondingly calculate the correlations between the time series of the stock daily returns, for each market separately. To obtain a better understanding of the dynamics of correlations in each market, a sliding window approach is used to calculate the market intra-correlations, using a 22-day window. In Figure 3 we present the dynamics of the intra-correlations for each of the six markets (see Figure S1 for the dynamics of the intra partial correlations for each of the six markets).

For each market, a bursting behavior for the intra-correlations is observed. This is consistent with previous findings [6]. Furthermore, a similarity in the appearance of intra-correlation bursts is noted for some of the markets as is elaborated in the next section.

**Figure 4:**
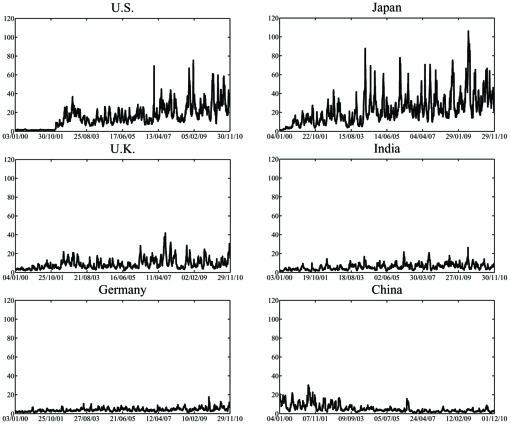
Dynamics of the ICF. The dynamics of the ICF for each market is plotted, for the period of 2000–2010.

Next, we calculate for each of the markets the Index Cohesive Force (ICF, Figure 4). High values of the ICF correspond to a state in which the market index dominates the behavior of the market, thus making it stiff and more prone to systematic failures. By studying Figure 4, it is possible to observe that some markets are similar in their dynamics of the ICF. Some similarities can be observed for the U.S., U.K., Germany, and Japan, whereas China shows a significantly different behavior. The ICF of the U.S. and Japan displays similarity in trend and magnitude, whereas U.K., India and Germany have a similar trend but much lower magnitude. Finally, China shows very different behavior than all other markets.

Markets featuring similar values of the ICF will have a similar dependency on the market index. Thus, if the indices of these markets are highly correlated, these markets should be strongly coupled. As such, the ICF provides new important information on these couplings.

### Inter-market correlations in the global financial market

The observed similarities of indices and correlation patters leads to the question of how synchronized stock markets are with respect to changes in these measures. Thus, we computed the meta-correlations – the correlations between the intra-correlations, using a 66-day window. The index correlations, the index volatility correlations and the ICF correlations were calculated using the same window size.

According to the index correlations the three “western” markets - U.S., U.K. and Germany - are highly correlated. The index correlations between Japan and India and all other markets are significantly weaker (the difference between these two groups is even more visible for the index volatility). China finally seems rather uncorrelated with the rest of the world, although some upward trend is visible (see a year by year breakdown for the market pair correlations in Figure S2, S3, S4, and S5). However, index correlations capture only partially the inter relations between different markets.

The introduction of the Index Cohesive Force makes things easier when one is considering the dynamics of a particular market and especially if one is interested in its stability, and provides a valuable measure to assess the state of each individual market. Previous work has shown that in the case of the U.S. market, low values of the ICF (lower than 10) correspond to a relatively healthy state of the market [3]. Specifically, it was found for the U.S. market that the ICF was close to zero, whereas in 2008 it was approaching 60. To further illustrate this point, we have found that the distribution of the ICF values change during times of economic stress, displaying much fatter tails (see example for the Japanese market, Figure S6). However, the ICF has been found to be highly fluctuating, and thus the ICF correlations between markets do not provide a reliable measure of the market inter relations.

**Figure 5:**
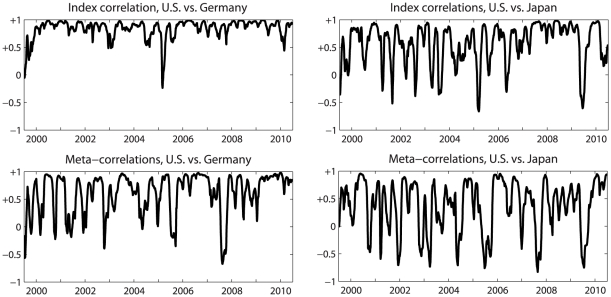
Index correlations (top) and Meta-correlations (bottom) for the U.S vs. Germany (left) and Japan (right). Both calculated using a 66-day window. The U.S. and Germany show a higher similarity for both measures than the U.S. and Japan. While both measures fluctuate over time, we observe that high correlations do not necessarily show jointly in the top and the bottom figure. We can thus differentiate between times of identical price movements (high index correlation) and global stress (high index correlation and high meta-correlation).

Much better results are obtained using the meta-correlations. Using this measure, we found that the three “western” markets have a high level of uniformity. The Japanese market appears to be significantly more influenced by the “west” than the Indian market, the Chinese has the lowest correlations. The latter is in line with what was expected for example given the capital controls and regulations in China and limitations for foreign investors [22]. Using a cross-correlation analysis, it is possible to further investigate the level of synchronization between the different markets. Typically, the lag (the time delay for maximum cross correlation) is 0 for high correlations and it fluctuates for low correlations (see Figure 5 for the correlations of the U.S., Germany and Japan and the Supplementary Figures S7, S8, S9, S10, S11, S12, S13, S14, S15, and S16 for the meta and index correlations for all markets). Generally speaking, we observe that the magnitude of inter market correlations fluctuates similarly like the magnitude of the intra-market correlations of the different market pairs.

### Dynamics of the global financial village

**Figure 6:**
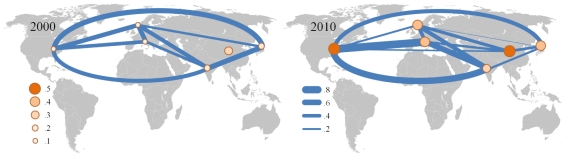
The global financial village for the years 2000 (left) and 2010 (right). The width of the edges of the graph is proportional to the meta-correlation between the markets it connects (right legend). The node size is proportional to the inter-correlations (left legend). For 2000 we observe markets with low intra-correlations and inter-correlations of similar magnitude, excluding China. For 2010 we observe much higher intra-correlations in all markets and a denser network of interdependencies. (The nodes for the U.K. and Germany are further away from each other than their geographical position).

The coupling of markets, as quantified by the meta-correlation, changes over time. The Japanese market switches between following the “western” and following the “eastern” worlds: for some time intervals it behaves very similar to the U.S. market (which is also similar to the U.K. and Germany markets), and at other times, the intra-correlations of Japan behave more similar to that of the Asian countries. Similar observations can be made for U.K. and Germany and their similarity to the U.S. vs. Asia. The interdependencies between India and China and the more developed markets are very volatile over time and show maxima in years with important global events (2001: 9/11-attacks, 2003: Iraq war, SARS, etc.). To illustrate the general development, we show the differences in coupling between markets during 2001 and 2010 (see Figure 6). The line strength is proportional to the level of the meta-correlations. The world's financial markets show a higher uniformity in the later years of our analysis. A video visualizing the development of market interdependencies is available as part of the Supplementary Information: http://dl.dropbox.com/u/16978699/globalmarket.mp4

## Discussion

This paper presents a new framework for quantitative assessments of the coupling and interdependences between different markets in the global financial village. The new approach also provides the means to study feedback between the micro (intra market) and the macro (inter markets) levels. More specifically, the stock-stock correlations in the individual markets represent local market dynamics, whereas the meta-correlations represent global market dynamics. Thus, the methodology presented here of intra and meta correlation analysis provides the means to study the bottom-top and top-down feedback mechanisms which take place in the world's economies.

Our results provide new information about the uniformity preset in the world's economies. We find significant uniformity for the three western markets, whereas Japan and India display a greater extent of multiformity; however, this multiformity is time dependent, and periods of significant uniformity with the western markets are observed. Unlike these, the case of China is significantly different. For all our measures it shows an amount of segmentation that is not in line with China's important role in the world economy, especially the huge trade flows we observe.

Earlier studies hint that the sole legal possibility to invest in emerging markets is not sufficient for their full integration with other markets. Investment funds and other institutional investors need accompanying financial products (i.e. country funds, depository receipts, and other derivatives), which are only gradually becoming available in emerging markets. Country specific risks, taxes and holding time requirements can further dampen cross-border investments [23], [24]. In China, significant parts of the economy are still state-owned. Furthermore, the Chinese stock market is differentiated into one for foreign investors and one for domestic investors. The amount of share holdings for private and foreign investors is still subject to substantial governmental restrictions [25]. These restrictions are obviously an effective and significant measure to deter foreign investors, and cause a partial de-coupling from other markets. Nevertheless, the methods presented here provide the means to quantify functional differences between developed and emerging markets. Further research is required to expand on this, especially by means of studying many more developed and emerging markets, and their coupling and interdependencies.

Finally, some interesting observations can be made about the general development of financial markets. It has been much debated that markets have become more coupled over the last years, and that we are observing the downside of this development right now during the debt crisis within the Eurozone and the U.S., expressed in pronounced synchronized movements of stock markets. From our analysis it becomes evident that this uniformity does not only stem from an increase of correlation between markets, but that there has also been an ongoing simultaneous shift towards uniformity in each single market.

In conclusion, using new specially devised analysis methods, we provide the means to investigate and quantify uniformity and multiformity in the global market, and changes in these measures. In the current era, when the global financial village is highly prone to systemic collapses which can sweep the entire village, our approach can provide a sensitive “financial seismograph” to detect early signs of global crises.

## Supporting Information

Figure S1 Dynamics of the intra partial correlation. For each market, we use a 22-day window, and in each window calculate the intra partial correlation, removing the effect of the index. Each horizontal line represents the average correlation of one stock (the left ordinate displays the number of the stock).
(TIF)

Figure S2 Average correlation of price indices, on a year-by-year basis. The year-by-year correlations were calculated as the average over all 66 day windows of each year. The pairs are sorted in descending order by total average correlation. Averaging by years allows us to judge on the general – medium to long run – interdependence between markets.
(EPS)

Figure S3 Average correlation of ICF, on a year-by-year basis. The year-by-year correlations were calculated as the average over all 66 day windows of each year. The pairs are sorted in descending order by total average correlation. Averaging by years allows us to judge on the general – medium to long run – interdependence between markets.
(EPS)

Figure S4 Average meta-correlation, on a year-by-year basis. The year-by-year correlations were calculated as the average over all 66 day windows of each year. The pairs are sorted in descending order by total average correlation. Averaging by years allows us to judge on the general – medium to long run – interdependence between markets.
(EPS)

Figure S5 Average index volatility correlation, on a year-by-year basis. The year-by-year correlations were calculated as the average over all 66 day windows of each year. The pairs are sorted in descending order by total average correlation. Averaging by years allows us to judge on the general – medium to long run – interdependence between markets.
(EPS)

Figure S6 Distributions of the Index Cohesive Force (ICF) values for the Japaneses market in different periods - 2000–2003 (blue), 2004–2007 (orange), and 2008–2010 (red). It is observable that the distributions are different for the studied periods, and that the ICF values are higher with a fat tail distribution for periods marked by strong economic fluctuations.
(TIFF)

Figure S7 Index correlations (top) and meta-correlations (bottom) for US-Germany and US-Japan, Lag with maximum correlation (blue cross) and correlation at lag 0 for the cross-correlation of the indices. Both performed for a 66-day window.
(EPS)

Figure S8 Index correlations (top) and meta-correlations (bottom) for US-UK and US-India, Lag with maximum correlation (blue cross) and correlation at lag 0 for the cross-correlation of the indices. Both performed for a 66-day window.
(EPS)

Figure S9 Index correlations (top) and meta-correlations (bottom) for US-China and UK-Germany, Lag with maximum correlation (blue cross) and correlation at lag 0 for the cross-correlation of the indices. Both performed for a 66-day window.
(EPS)

Figure S10 Index correlations (top) and meta-correlations (bottom) for UK-Japan and UK-India, Lag with maximum correlation (blue cross) and correlation at lag 0 for the cross-correlation of the indices. Both performed for a 66-day window.
(EPS)

Figure S11 Index correlations (top) and meta-correlations (bottom) for UK-China and Germany-Japan, Lag with maximum correlation (blue cross) and correlation at lag 0 for the cross-correlation of the indices. Both performed for a 66-day window.
(EPS)

Figure S12 Index correlations (top) and meta-correlations (bottom) for Germany-India and Germany-China, Lag with maximum correlation (blue cross) and correlation at lag 0 for the cross-correlation of the indices. Both performed for a 66-day window.
(EPS)

Figure S13 Index correlations (top) and meta-correlations (bottom) for Japan-India and Japan-China, Lag with maximum correlation (blue cross) and correlation at lag 0 for the cross-correlation of the indices. Both performed for a 66-day window.
(EPS)

Figure S14 Index correlations (top) and meta-correlations (bottom) for India-China, Lag with maximum correlation (blue cross) and correlation at lag 0 for the cross-correlation of the indices. Both performed for a 66-day window.
(EPS)

Figure S15 Cross-correlation plot of meta-correlations. Black marker on maximum if the correlation is higher than .7, U.S. against all other markets. Performed for a 66-day window.
(EPS)

Figure S16 Cross-correlation plot of index correlations. Black marker on maximum if the correlation is higher than .7, U.S. against all other markets. Performed for a 66 day window.
(EPS)
