# Supplementary figures and images for: Evolvement of Uniformity and Volatility in the Stressed Global Financial Village
Source: PLoS One. 2012 Feb 8;7(2):e31144. doi: 10.1371/journal.pone.0031144 (PMC3275621; doi:10.1371/journal.pone.0031144)

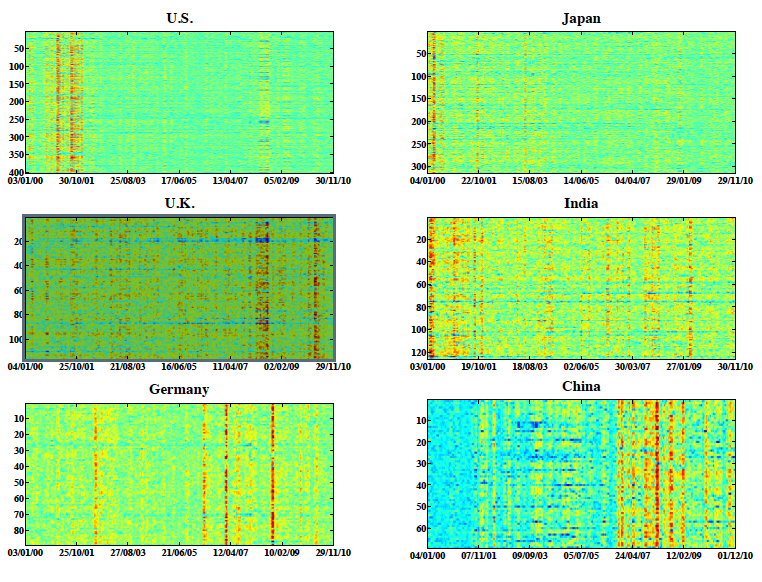

Supplement: Figure S1 — Dynamics of the intra partial correlation. For each market, we use a 22-day window, and in each window calculate the intra partial correlation, removing the effect of the index. Each horizontal line represents the average correlation of one stock (the left ordinate displays the number of the stock). (TIF) [file pone.0031144.s001.tif]

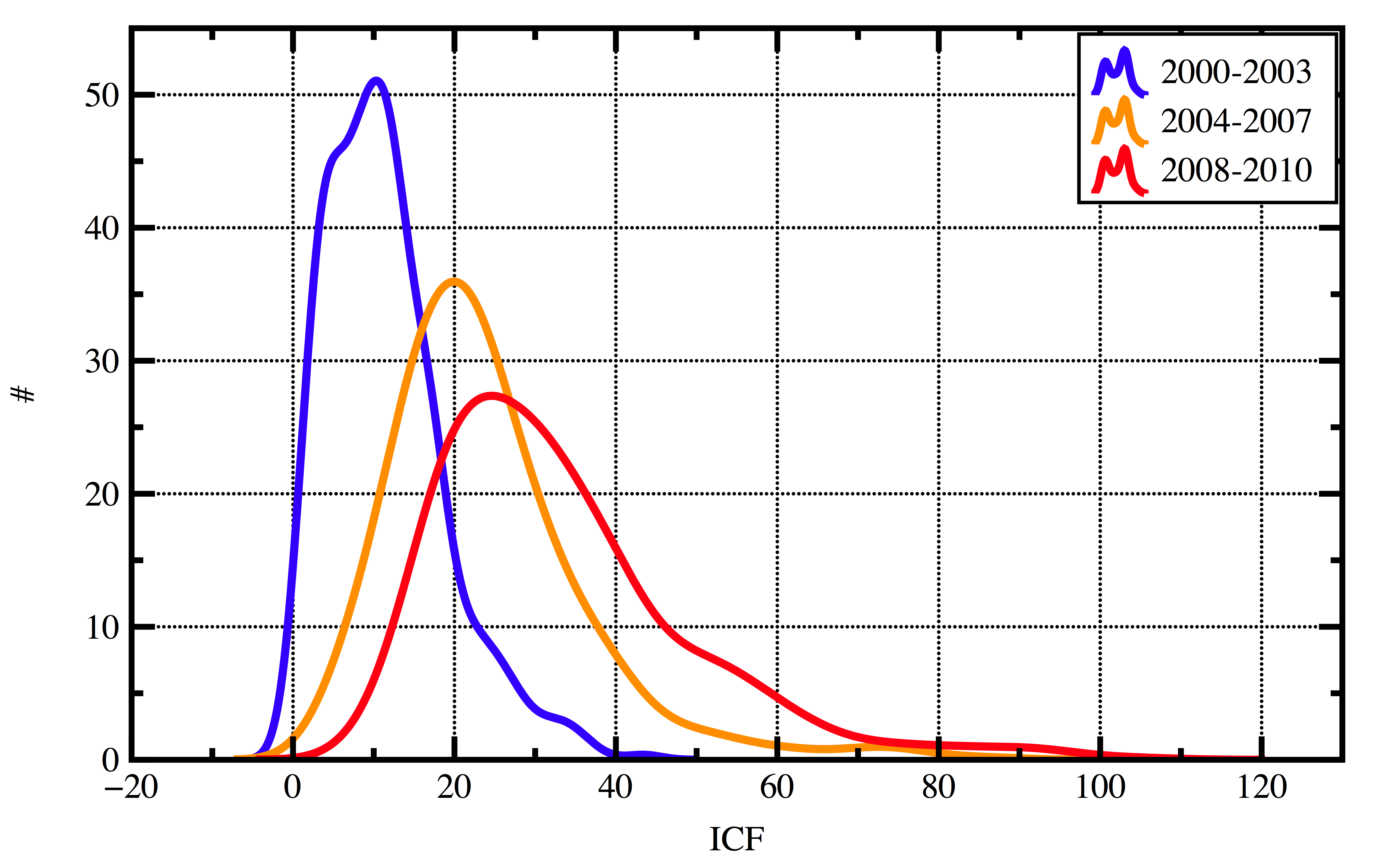

Supplement: Figure S6 — Distributions of the Index Cohesive Force (ICF) values for the Japaneses market in different periods - 2000–2003 (blue), 2004–2007 (orange), and 2008–2010 (red). It is observable that the distributions are different for the studied periods, and that the ICF values are higher with a fat tail distribution for periods marked by strong economic fluctuations. (TIFF) [file pone.0031144.s006.tif]
